# Novel Technique for Retinal Nerve Cell Regeneration with Electrophysiological Functions Using Human Iris-Derived iPS Cells

**DOI:** 10.3390/cells10040743

**Published:** 2021-03-28

**Authors:** Naoki Yamamoto, Noriko Hiramatsu, Mahito Ohkuma, Natsuko Hatsusaka, Shun Takeda, Noriaki Nagai, Ei-ichi Miyachi, Masashi Kondo, Kazuyoshi Imaizumi, Masayuki Horiguchi, Eri Kubo, Hiroshi Sasaki

**Affiliations:** 1 Department of Ophthalmology, Kanazawa Medical University, Ishikawa 920-0293, Japan; n-hatsu@kanazawa-med.ac.jp (N.H.); s-takeda@kanazawa-med.ac.jp (S.T.); kuboe@kanazawa-med.ac.jp (E.K.); mogu@kanazawa-med.ac.jp (H.S.); 2 Graduate School of Health Sciences, Fujita Health University, Aichi 470-1192, Japan; 3 Research Promotion and Support Headquarters, Fujita Health University, Aichi 470-1192, Japan; norikoh@fujita-hu.ac.jp; 4 Department of Physiology, School of Medicine, Fujita Health University, Aichi 470-1192, Japan; m-ohkuma@fujita-hu.ac.jp (M.O.); emiyachi@fujita-hu.ac.jp (E.-i.M.); 5 Faculty of Pharmacy, Kindai University, Osaka 577-8502, Japan; nagai_n@phar.kindai.ac.jp; 6 Department of Food Science and Nutrition, Nagoya Women’s University, Aichi 467-8610, Japan; 7 Department of Respiratory Medicine, School of Medicine, Fujita Health University, Aichi 470-1192, Japan; mkond@fujita-hu.ac.jp (M.K.); jeanluc@fujita-hu.ac.jp (K.I.); 8 Department of Ophthalmology, School of Medicine, Fujita Health University, Aichi 470-1192, Japan; masayuki@fujita-hu.ac.jp

**Keywords:** human iris tissue stem/progenitor cells, human iris-derived iPS cells, p75NTR, recoverin, retinal ganglion cell, electrophysiology

## Abstract

Regenerative medicine in ophthalmology that uses induced pluripotent stem cells (iPS) cells has been described, but those studies used iPS cells derived from fibroblasts. Here, we generated iPS cells derived from iris cells that develop from the same inner layer of the optic cup as the retina, to regenerate retinal nerves. We first identified cells positive for p75NTR, a marker of retinal tissue stem and progenitor cells, in human iris tissue. We then reprogrammed the cultured p75NTR-positive iris tissue stem/progenitor (H-iris stem/progenitor) cells to create iris-derived iPS (H-iris iPS) cells for the first time. These cells were positive for iPS cell markers and showed pluripotency to differentiate into three germ layers. When H-iris iPS cells were pre-differentiated into neural stem/progenitor cells, not all cells became positive for neural stem/progenitor and nerve cell markers. When these cells were pre-differentiated into neural stem/progenitor cells, sorted with p75NTR, and used as a medium for differentiating into retinal nerve cells, the cells differentiated into Recoverin-positive cells with electrophysiological functions. In a different medium, H-iris iPS cells differentiated into retinal ganglion cell marker-positive cells with electrophysiological functions. This is the first demonstration of H-iris iPS cells differentiating into retinal neurons that function physiologically as neurons.

## 1. Introduction

The human body is composed of approximately 3.72 × 10^13^ cells of about 270 types, as estimated in 2013 by Bianconi et al. using bibliographical and/or mathematical approaches [1]. Until 2000, the basic research conducted to differentiate these various cell types in vitro used mainly embryonic stem (ES) cells (Table 1). In 1981, Dr. M. Evans and Dr. M. Kaufman created ES cells derived from the inner cell mass of mouse blastocysts [2]. Embryonic stem cells of various other mammals were then produced, and in 1998, Prof. James Thomson succeeded in producing human ES cells [3]. Human ES cells can be cultured and proliferated indefinitely in an undifferentiated state when they are cultured under special culture conditions. The injection of human ES cells into mouse muscle tissue results in a teratoma, with the desired neuroepithelium, smooth muscle, bone cells, etc., forming inside the tumor. Since cells such as cartilage and intestinal epithelium were produced by this process, the cells had “pluripotency”, which allowed them to differentiate into various cells. However, human ES cells are “non-self” cells because they are produced by destroying fertilized human eggs, and they have genetic information and traits derived from the blastocysts used in the production of the ES cells. Moreover, in the case of transplantation to another person, there is a risk of immune rejection.

In March 2009, U.S. President Barack Obama ended the ban against the use of federal funding for human ES cell research, accelerating the impetus for research using ES cells. In Japan, the double examination system for research using human ES cells was also abolished, leaving the ethical aspects of research using human ES cells to the ethics committee of each research institution. For various reasons, the use of ES cells had been limited to basic research, as there have been ethical issues associated with breaking and separating fertilized eggs. However, in August 2006, Professor Shinya Yamanaka of Kyoto University succeeded in creating induced pluripotent stem (iPS) cells by introducing several types of genes into the skin cells of adult mice [4]. In November 2007, iPS cells derived from human cells were successfully produced [5]. Following on this success, “regenerative medicine” with transplanted iPS cells has begun to draw much attention as a new approach for restoring the function of cells and tissues damaged by injury or disease (Table 2).

Worldwide, the estimated number of individuals with retinitis pigmentosa, an eye disease that causes blindness, is over 1.5 million [6,7]. In Japan, glaucoma is the leading cause of visual impairment, followed by retinitis pigmentosa and diabetic retinopathy; in addition, the proportion of all visual impairment attributable to glaucoma is increasing [8]. With current treatments (e.g., eye surgery), it is difficult to completely restore visual acuity, and investigations of retinal regeneration have thus begun to examine the use of new transplanted cells instead of injured retinal neurons to regenerate retinal function by reconstructing a network with the cells remaining in the host retina. Such retinal regeneration research has included studies using neural stem cells derived from tissues other than eyeball tissue [9,10,11,12,13], research using tissues (cells) that compose the eyeball [14,15,16,17,18,19,20], and basic studies using ES and iPS cells [21,22,23]. In September 2014, patients with age-related macular degeneration (AMD) were successfully transplanted with an autologous transplant sheet made from induced pluripotent stem cell-derived retinal pigment epithelial (iPSC-RPE) cells [24].

iPS cells are essentially produced from skin fibroblasts. Our group is currently focusing on tissue regeneration in the field of ophthalmology, especially the regeneration of retinal nerve cells. To obtain cells for regenerating the retina, in the present study we focused on iris tissue, since iris self-tissue can be safely collected in the process of disease treatment. The reasons for the choice of iris tissue were as follows: (1) In the embryological classification, iris cells are included in the same inner layer of the optic cup as retinal neurons; (2) The crystalline lens of the newt is prone to transdifferentiation, and the lens can thus be regenerated from the iris dorsally when artificially excised [25,26,27]; (3) The cytokine gene expression profile is similar between retinal pigment epithelial cells and iris pigment epithelial cells [28]; (4) Partial iris resection has been established as a treatment for angle-closure glaucoma in humans, in which a part of the iris is excised and the atrioventricular canal is reconstructed (making it possible to collect iris self-tissue safely and reliably).

In our prior research, we used positivity for p75NTR (CD271) as a marker for neural stem/progenitor cells in mouse iris tissue, and we observed that p75NTR-positive cells efficiently differentiated into cells expressing photoreceptor markers [29]. In the present study, we created human iris-derived iPS (H-iris iPS) cells for the first time by using iris tissue stem cells (H-iris stem/progenitor cells) selected by p75NTR, and we examined the differentiation of these iPS cells into Recoverin-positive (photoreceptor-like) cells expressing Recoverin and ganglion cells expressing Neurofilament-M and Brn-3b. The differentiated cells were verified to be physiologically functioning cells by an analysis of their electrophysiology.

## 2. Materials and Methods

### 2.1. Immunohistochemistry of Human Iris Tissue

The tissues used for the immunohistochemistry were collected in the course of treating glaucoma patients by partial iris resection. This study was performed with the approval (No. 05-065) of the Ethics Review Committee of Fujita Health University. All subjects provided written informed consent for their tissue to be used, and the study complies with the tenets of the Declaration of Helsinki for research involving human tissue.

A piece of human iris tissue was fixed in SUPER FIX™ rapid fixative solution (Kurabo Industries, Osaka, Japan) [29]. Paraffin sections were prepared from the fixed human iris tissue in the usual manner and incubated with anti-p75NTR polyclonal antibody (1:200; Alomone Labs, Jerusalem, Israel) for 1 h at 37 °C. The secondary antibodies were incubated with Alexa Fluor^®^ 594-labeled antibody (1:1000; Invitrogen, Carlsbad, CA, USA) for 1 h at 37 °C. DAPI (Vectashield H-1200; Vector Laboratories, Burlingame, CA, USA) was used for nuclear staining. A fluorescence microscope (Power BX-51; Olympus, Tokyo, Japan) was used for observation. Hematoxylin and eosin (HE) staining was also performed on the continuous sections.

### 2.2. Cell Isolation and Culturing of Human Iris Tissue

Human iris tissue was processed as described [29,30]. Briefly, iris tissue was treated with 0.2% collagenase (Sigma-Aldrich, St. Louis, MO, USA) and washed twice with phosphate-buffered saline solution (PBS; Sigma). The isolated cells were cultured in iris culture medium (iris medium): Advanced Dulbecco’s Modified Eagle Medium/Ham’s F12 (Advanced DMEM/F12; ThermoFisher Scientific, Waltham, MA, USA) supplemented with 5% mixed serum (heat-inactivated human serum [Sigma], KnockOut™ Serum Replacement [ThermoFisher Scientific], and Artificial Serum, Xeno-free [Cell Science & Technology Institute, Miyagi, Japan] in a 5:3:2 ratio), 10 ng/mL basic fibroblastic growth factor (b-FGF; Sigma), 10 ng/mL epidermal growth factor (EGF; Sigma), ×50 GlutaMAX (Gibco Invitrogen, Carlsbad, CA, USA), CultureSure^®^ Y-27632 solution (only for the beginning of the culture; Fujifilm Wako Pure Chemical Corp., Osaka, Japan), and 1% penicillin/streptomycin (Sigma) in a 35 mm culture dish coated with collagen type-1 (Toyobo, Osaka, Japan) at 37 °C in a 5% CO_2_ humidified incubator.

### 2.3. Cell Sorting of Iris Tissue Stem/Progenitor Cells

p75NTR has been shown to be an effective marker for iris tissue stem/progenitor cells [29], and we thus used p75NTR for the cell sorting. The human iris-derived culture cells were subcultured using TrypLE™ Select (Invitrogen), then washed twice with PBS. Anti-p75NTR polyclonal antibody (1:200) was added and allowed to incubate at 4 °C for 30 min. The cells were washed with PBS and incubated with Alexa Fluor^®^ 488-labeled antibody (1:1000; Invitrogen) at 4 °C for 30 min. The p75NTR-Alexa Fluor^®^ 488-labeled cells were sorted (FACSVantage SE; BD Biosciences, San Jose, CA, USA) for the selection of the p75NTR-positive cells. To remove the dead cells from the sorting, 1 μg/mL of propidium iodide (PI; ThermoFisher Scientific) was added to the samples.

### 2.4. Preparation and Verification Experiment of iPS Cells

We prepared H-iris iPS cells by cell reprogramming using human iris tissue-derived p75NTR-positive stem/progenitor cells (H-iris stem/progenitor cells). The H-iris stem/progenitor cells were reprogrammed by a micro-electroporation method using an Epi5™ Episomal iPSC Reprogramming Kit (ThermoFisher Scientific) [31,32]. The reagent concentration of the kit was 2 μL/well (2 μg), and the concentration of the introduced acceleration reagent was also 2 μL/well (2 μg). At the first stage, Geltrex^®^ (Life Technologies, Carlsbad, CA, USA) for the coating of the dish and Essential 8™ Medium (ThermoFisher Scientific) as the iPS culture medium were used. After the 10th generation, StemFit^®^ and iMatrix-511 (Laminin-5; Takara Bio, Shiga, Japan) were used as the iPS culture medium and the coating agent, respectively. The experiment was carried out with the approval (No. 232) of the Recombinant DNA Experiment Committee of Fujita Medical University.

The iPS cells were verified as follows. Some fixed cells were stained with alkaline phosphatase (ALP), which is a commonly used marker that is highly expressed in all pluripotent stem cells, including ES cells and iPS cells, with a Red-Color™ AP Staining Kit (System Biosciences, Palo Alto, CA, USA) following the manufacturer’s protocol, then observed under a microscope [33].

Immunofluorescence staining was performed as described [33]. Briefly, some fixed cells were treated with 0.5% Triton^®^ X-100 (Fujifilm Wako) for 5 min, and then Protein Block Serum-Free Ready-To-Use (Agilent Technologies, Santa Clara, CA, USA) was added for blocking for 5 min at room temperature. One of the following was added as a primary antibody to PBS containing 1% albumin: anti-human SOX2 (an intranuclear iPS marker protein) monoclonal antibody (1:100; ThermoFisher Scientific), anti-human OCT3/4 (an intranuclear iPS marker protein) polyclonal antibody (1:500; Medical & Biological Laboratories, Aichi, Japan), or anti-human SSEA-4 (an iPS cytoplasmic protein marker) monoclonal antibody (1:100; Abcam, Cambridge, UK). Alexa Fluor^®^ 594-labeled antibody (1:500; ThermoFisher Scientific) was added as a secondary antibody. DAPI (Vectashield H-1200; Vector Laboratories) was used for nuclear staining. The immunostaining was evaluated using a fluorescence microscope (Power IX-71 and DP-71; Olympus).

In addition, the cell surface marker SSEA-4 was verified by a flow cytometry (FCM) analysis. Anti-human SSEA-4 monoclonal antibody was added to the cells as the primary antibody, and then Alexa Fluor^®^ 488 (1:500; ThermoFisher Scientific) was added as the secondary antibody, and the mixture was incubated. A FACSCan (Becton Dickinson and Company, Franklin Lakes, NJ, USA) was used for the cytometric analysis. Propidium iodide (PI) was added to the samples to remove the dead cells from the sorting.

Cloning was performed by passage with a small number of seeded cells. A gene expression analysis of iPS cells was performed using a TaqMan^®^ quantitative real-time polymerase chain reaction (qRT-PCR) as described [33]. Briefly, a TaqMan^®^ Gene Expression Cells-to-CT™ Kit (ThermoFisher Scientific) was used for the extraction of total RNA from cells and the reverse transcription reaction. The cDNA was then used as a template for PCR amplification. For the TaqMan^®^ real-time PCR (qPCR) assays, the qPCR was performed using an ABI PRISM 7900 HT Sequence Detection System (ThermoFisher Scientific). A primer and a probe for the iPS cell-marker genes, i.e., *SOX2* (Hs00415716_m1), *OCT3/4* (Hs00742896_s1), *NANOG* (Hs04260366_g1) and *KLF4* (Hs00358836_m1) were used, and *GAPDH* (Hs99999905_m1) was used for endogenous control.

### 2.5. Formation of Teratomas and Embryoid Bodies

H-iris iPS cells were subcutaneously transplanted by injection into immunocompromised mice (KSN-nu/Slc, Japan SLC, Shizuoka, Japan) to test teratoma formation in vivo, as described [5]. All procedures were performed according to the ARVO Statement for the Use of Animals in Ophthalmic and Vision Research and were approved (No. M2701) by the Education and Research Center for Animal Models of Human Diseases of Fujita Health University. Teratoma formation was observed 9 weeks after injection. In the histological examination, paraffin sections were prepared by the usual method, stained with HE, and observed under a microscope.

In order to confirm the pluripotency of the gene-transferred cells, we attempted to generate embryoid bodies (EBs) and teratomas. The EBs were prepared by culturing as described [33], but with the addition of 0.3% methylcellulose (Sigma). The EBs were collected from the floating cultures on day 10 and then were treated by the cell block method for the preparation of section specimens. The section specimens were HE-stained and then immunostained with the following primary antibodies: anti-human Tubulin-β3 monoclonal antibody (1:100; BioLegend, San Diego, CA, USA) as an ectoderm marker protein, anti-human alpha smooth muscle actin (α-SMA) polyclonal antibody (1:100; Abcam) as a mesoderm marker protein, and anti-human α-Fetoprotein (AFP) polyclonal antibody (Proteintech Group, Rosemont, IL, USA) as an endoderm marker protein. As a secondary antibody, Histofine^®^ Simple Stain™ MAX-PO MULTI (Nichirei, Tokyo) was added. We used the Liquid 3,3′-Diaminobenzidine Tetrahydrochloride (DAB)+ Substrate Chromogen System (Dako Omnis; Agilent Technologies) as a colorimetric substrate, and the cell nuclei were stained with hematoxylin.

### 2.6. Differentiation into Nerve Cells and Recoverin-Positive Cells

H-iris iPS cells were induced to differentiate into neural stem/progenitor cells in a pre-differentiation medium by adhesive culture. For the iPS cell subculture, iPS cells were gently peeled from the dish and subcultured on a dish coated with Poly-L-Lysine (Sigma) and Laminin (BD Biosciences) so that the shape of the colony was maintained. The composition of the pre-differentiation medium was as follows: Neurobasal™ Medium (ThermoFisher Scientific) supplemented with 10 ng/mL b-FGF, 10 ng/mL EGF, ×100 N-2 MAX Media Supplement (R&D Systems, Minneapolis, MN, USA), ×50 GlutaMAX, and 1% penicillin/streptomycin. Next, the cells were cultured in a nerve cell differentiation medium. After sorting of the cells with p75NTR, the culture was supplemented with a nerve cell differentiation medium that consisted of pre-differentiation medium and 1 μM retinoic acid (RA, Sigma). Some of the cells were fixed with 4% paraformaldehyde, treated with 0.1% saponin solution to permeabilize the membranes, stained with p75NTR antibody and Nestin antibody, and analyzed by FCM. Another aliquot of the cells sorted by p75NTR were collected and observed with a fluorescence microscope. The cells were then cultured in photoreceptor differentiation medium. The composition of the photoreceptor differentiation medium was as follows: advanced DMEM/F12 supplemented with 2 μM RA, 200 μM butylated hydroxyanisole (BHA; Sigma), 10 ng/mL b-FGF, 10 ng/mL EGF, ×100 N-2 MAX Media Supplement, ×100 GlutaMAX, and 1% penicillin/streptomycin.

The neurons and photoreceptors were immunostained using the following primary antibodies: anti-Musashi monoclonal antibody (1:100; ThermoFisher Scientific), anti-human Nestin monoclonal antibody (1:100), anti-human Neurofilament-M polyclonal antibody (1:200), anti-human MAP2 monoclonal antibody (1:200), anti-human Neurofilament-H polyclonal antibody (1:200; Merck KGaA, Darmstadt, Germany), anti-human Tubulin-β3 monoclonal antibody, and anti-human Recoverin polyclonal antibody (1:500, Merck). DAPI was used for nuclear staining.

A primer and probe were used for one of the photoreceptor cell-marker genes, i.e., *Recoverin* (Hs00610056_m1). *GAPDH* (Hs99999905_m1) was used for endogenous control.

### 2.7. Differentiation into Retinal Ganglion Cells

The induction of differentiation into retinal ganglion cells was performed as described [34]. Briefly, cell aggregates were formed from neural stem/progenitor cells in suspension culture and then differentiated into ganglion cells. *TUBULIN-βIII* (Hs00801390_s1), *NEUROFILAMENT-M* (Hs00193572_m1), and *MAP2* (Hs00258900_m1), *BRN-3B* (Hs00231820_m1) were used. *GAPDH* (Hs99999905_m1) was used for endogenous control. An anti-human Brn-3b (retinal ganglion cell marker) monoclonal antibody (1:100; Santa Cruz Biotechnology, Dallas, TX, USA) was used as the primary antibody.

### 2.8. Electrophysiology

For patch-clamp recording, the recording culture dish was mounted on the stage of an inverted microscope (TMD300; Nikon, Tokyo). The indifferent electrode was an Ag-AgCl wire connected to the culture dish. Membrane voltages and currents were recorded in the whole-cell configuration using a patch-clamp amplifier (Axopatch 200B; Molecular Devices, San Jose, CA, USA) linked to a computer [35,36,37]. The voltage-clamp and current-clamp procedures were controlled by pCLAMP software (Molecular Devices). The data were low-pass filtered with a cut-off frequency of 5 kHz and then digitized at 10 kHz by an analog-to-digital interface (Digidata 1320A; Molecular Devices). Cultured cells were perfused at 1 mL/min with Ringer solution bubbled with 100% O_2_. The composition of HEPES-buffered Ringer solution (in mM) was 135 NaCl, 5 KCl, 2 CaCl2, 1 MgCl2, 10 glucose, and 10 HEPES (the pH was adjusted to 7.4 with KOH). The recording pipette was filled with pseudo-intracellular solution with the following composition (in mM): 140 mM KCl, 1 mM CaCl_2_, 2 mM MgCl_2_, 5 mM BAPTA, 10 mM HEPES. The solution was adjusted with KOH to pH 7.4. The pipette resistance was 6–8 MΩ. Tetrodotoxin (1 µM TTX, a voltage-gated sodium channel blocker) was applied through the bath.

## 3. Results

### 3.1. p75NTR-Positive Cells Observed in Human Iris Tissue and Cultured Cells

**Figure 1:**
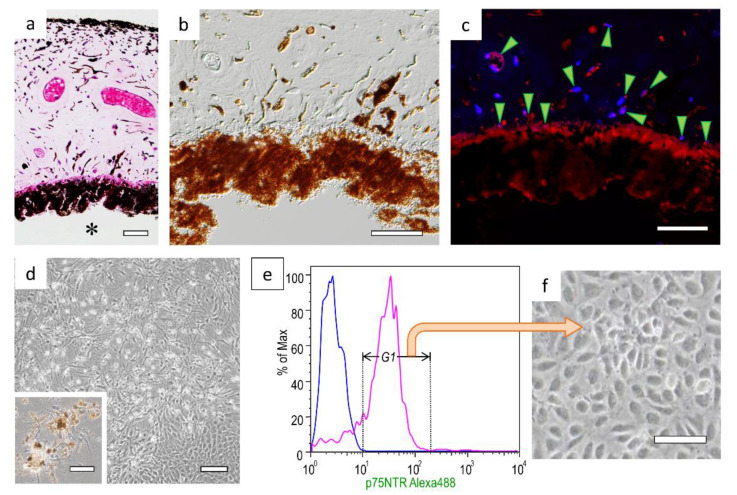
p75NTR-positive cells observed in human iris tissue and cultured cells: (**a**) HE-stained human iris tissue; * The lens side of the iris. (**b**) Differential interference contrast image of iris tissue. (**c**) Fluorescent immunostaining of p75NTR; *arrowheads* = p75NTR-positive cells. (**d**) Iris-derived cells were cultured for 10 days; the *inset* shows cells growing from around the pigmented cells on the third day of culture. (**e**) p75NTR-positive cells (region of Gate 1, G1) were isolated with a cell sorter; the *blue line* represents the histogram of negative cells. (**f**) Morphology of p75NTR-positive sorted cells. Bars in panels (**a**–**c**), 50 μm; bars in panel (**d**)’s *inset* and panel (**f**), 100 μm; bar in panel (**d**), 200 μm.

p75NTR-positive cells were observed in the parenchymal cells of human iris tissue and in the base layer of the iris pigment epithelial cells on the lens side (Figure 1a–c). When iris tissue was treated with enzymes and cultured, three types of cell morphology were observed: a fibroblast-like morphology, a nerve cell-like morphology with protrusions, and an epithelial cell-like morphology (Figure 1d). During culturing, melanin in the cytoplasm degranulated from the cells. Therefore, when cell sorting was performed using the cell surface marker p75NTR (Figure 1e), cells showing the epithelial cell-like morphology and containing no melanin granules were isolated (iris tissue stem/progenitor cells; Figure 1f).

### 3.2. Preparation and Verification of iPS Cells Using Human Iris Tissue Stem/Progenitor Cells

**Figure 2:**
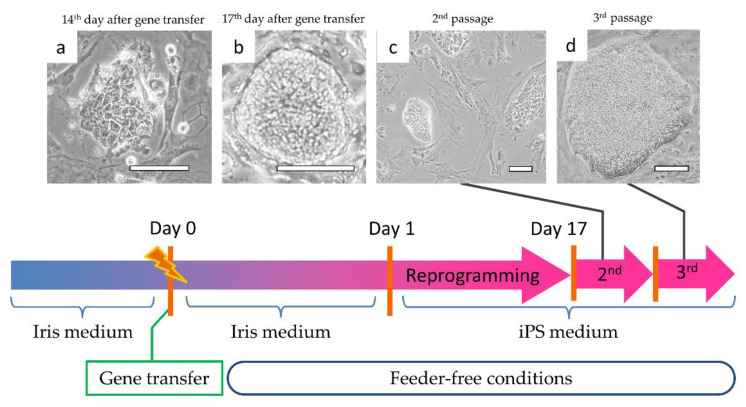
Reprogramming process of H-iris iPS cells. Cell morphology at the (**a**) 14th day of gene transfer; (**b**) 17th day of transfer; (**c**) second passage; (**d**) third passage. Bars: 100 μm.

Iris tissue stem/progenitor cells were reprogrammed using the Epi5™ Episomal iPSC Reprogramming Kit cited above, by a micro-electroporation method. The reprogramming protocol is illustrated in Figure 2. From around the 14th day of reprogramming, colonies with densely aggregated, proliferating cells were observed (Figure 2a). On the 17th of reprogramming, we peeled the cells off in order to surround the colony, and we replaced the cells (Figure 2b). In the second passage, a denser cell clump was observed (Figure 2c). As the passages continued, an aggregation of large iPS cells showing a colony-like morphology was observed (Figure 2d).

**Figure 3:**
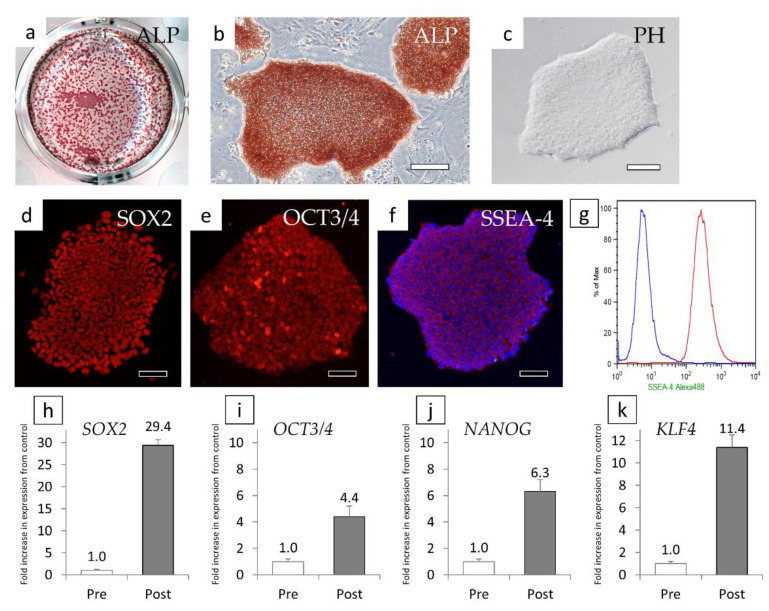
Verification of H-iris iPS cells. (**a**) Low magnification of an ALP-stained multiplate. (**b**) ALP staining of aggregated cell colonies. (**c**) The morphology of cloned colonies observed with a phase contrast microscope. (**a**–**f**) Fluorescent immunostaining of cloned colonies for (**d**) SOX2, (**e**) OCT3/4, and (**f**) SSEA-4. (**g**) Analysis of SSEA-4 by FCM. The *blue line* represents the histogram of negative cells. (**h**–**k**) Relative semi-quantitative analysis of gene expression of (**h**) *SOX2*, (**i**) *OCT3/4*, (**j**) *NANOG*, and (**k**) *KLF4*. Pre: iris tissue stem/progenitor cells before gene transfer, Post: cells of cloned colonies (Post). Bars: 100 μm.

When ALP staining was performed using cells passaged three times, only the cells forming agglutinated cell colonies were positive (Figure 3a,b). We thus attempted to clone cells for each colony, and colonies with good cell proliferation and uniform cell morphology were cloned (Figure 3c). Iris-derived cells that could not be reprogrammed remained around the colony before cloning (Figure 2d), but as a result of continued cloning and passage, they were unable to proliferate and were eliminated.

The cloned cells were immunostained and were observed to be SOX2-positive, OCT3/4-positive, and SSEA-4-positive (Figure 3d–f). When we examined the positivity for SSEA-4 (a cell surface protein) by FCM, we observed that almost all of the cells were SSEA-4-positive cells (Figure 3g). A relative semi-quantitative analysis of gene expression using qRT-PCR revealed that compared to the levels in iris tissue stem/progenitor cells before reprogramming, *SOX2* was increased by 29.4-fold (Figure 3h), *OCT3/4* was increased by 4.4-fold (Figure 3i), *NANOG* was increased by 6.3-fold (Figure 3j), and *KLF4* was increased by 11.4-fold (Figure 3k). Based on these results, the cloned cells showed the characteristics of iPS cells, and we thus changed the cell name to H-iris iPS cells.

### 3.3. Verification of Formed Teratomas and EBs

**Figure 4:**
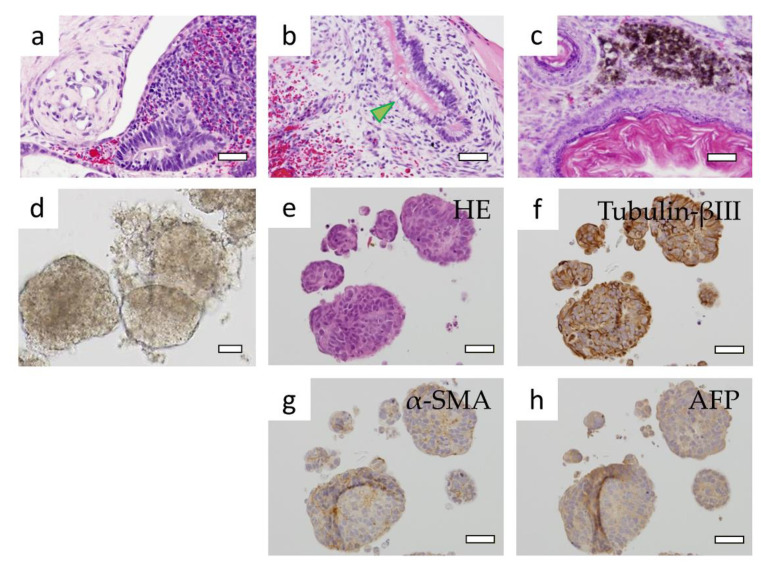
Observations of the formed teratomas and EBs. (**a**–**c**) The gastrointestinal tract (*arrowhead*), skin, and cells that produce melanin were mixed in the HE-stained sections of teratomas. (**d**) Morphology of suspension-cultured EBs. EB section specimens stained with (**e**) HE, (**f**) Tubulin-βIII, (**g**) α-SMA, and (**h**) AFP. Bars: 50 μm.

Teratoma formation was observed 9 weeks after transplantation into the mice. When the tissue sections of HE-stained teratomas were observed, they showed different germ types of cell morphologies despite their proximity, and some of them resembled tissue of the gastrointestinal tract with mucus in the cytoplasm. We also observed another region with an arrangement of keratinocytes and keratinized layers resembling skin, and other cells containing melanin (Figure 4a–c).

H-iris iPS cells that were suspension-cultured in a medium containing 0.3% methylcellulose formed cell aggregates (Figure 4d,e). When the section specimen was immunostained, Tubulin-βIII was most strongly expressed, and cells of different regions were positive for α-SMA and AFP (Figure 4f–h). These results confirmed that H-iris iPS cells form teratomas and have pluripotency to differentiate into three germ layers.

### 3.4. Differentiation into Nerve Cells and Recoverin-Positive Cells

**Figure 5:**
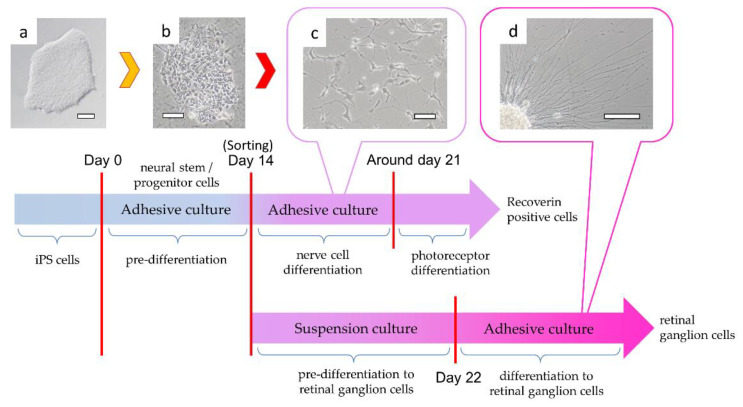
Process of differentiation of H-iris iPS cells to nervous system cells. Cells were pre-differentiated from (**a**) H-iris iPS cells to (**b**) neural stem/progenitor cells by adhesive culture. (**c**) Cells sorted with p75NTR were differentiated into neurons. By changing the medium condition, the nerve cells were differentiated into Recoverin-positive cells. (**d**) In contrast, after suspension culture, neurites were elongated by adhesive culture and differentiated into retinal ganglion cells. Bars: 100 μm.

**Figure 6:**
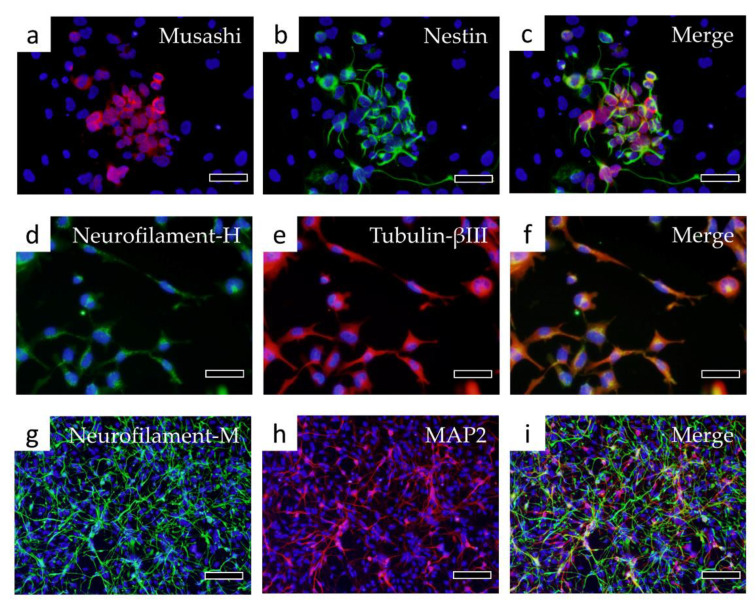
Immunostaining of cells differentiated into nerve cells. Fluorescent immunostaining was performed with the following markers of neural stem/progenitor cells: (**a**) Musashi, (**b**) Nestin, (**c**) Merge of Musashi and Nestin. Immunostaining was then performed with the following markers of nerve cells: (**d**) Neurofilament-H, (**e**) Tubulin-βIII, (**f**) Merge of Neurofilament-H and Tubulin-βIII, (**g**) Neurofilament-M, (**h**) MAP2, (**i**) Merge of Neurofilament-M and MAP2. Bars in panels (**a**–**f**): 50 μm, bars in panels (**g**–**i**): 100 μm.

**Figure 7:**
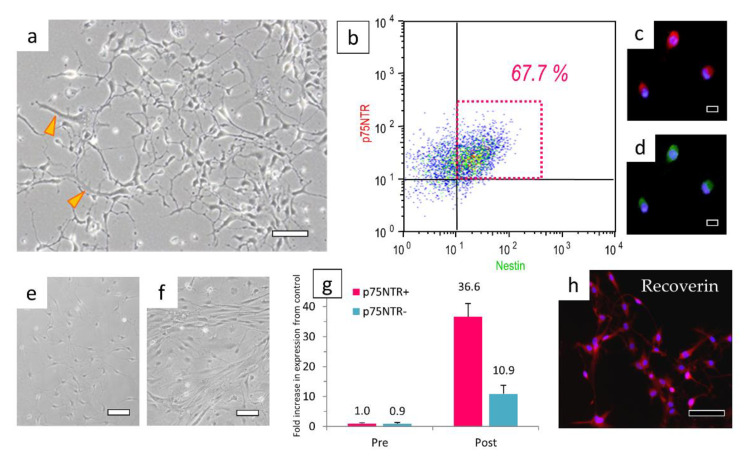
Cells sorted with p75NTR can concentrate Recoverin-positive cells. (**a**) Before p75NTR sorting, cells with large cytoplasms (*arrowheads*) were mixed. (**b**) Fixed cells were analyzed for p75NTR and Nestin by FCM. Most of the cells sorted in the strong positive region of p75NTR were double-positive for (**c**) p75NTR and (**d**) Nestin. (**e**,**f**) The morphology of cells sorted by their strong positivity for (**e**) p75NTR or (**f**) their weak positivity or negativity for p75NTR. (**g**) Relative semi-quantitative analysis of *Recoverin* gene expression in cells selected by p75NTR, and pre/post-differentiated cells. (**h**) Fluorescent immunostaining of Recoverin in differentiated cells. Bar in panel (**a**): 50 μm, bars in panels (**c**,**d**): 20 μm, bars in panels (**e**,**f**,**h**): 100 μm.

H-iris iPS cells were differentiated into neural stem/progenitor cells by pre-differentiation medium in adhesive culture. In those cells, rather than the colonies of iPS cells, the contours of individual cells could be clearly confirmed (Figure 5a,b). After 14 days of culture in pre-differentiation medium, some cells differentiated into Musashi and Nestin-positive neural stem/progenitor cells (Figure 6a–c). When continuously cultured in a nerve differentiation medium, a large number of cells with extended protrusions of nerve-like cells were observed. However, some cells had a large cytoplasm, and some cells did not show the morphology of nerve-like cells (Figure 5c). In the cell immunostaining, many cells were double-positive for Neurofilament-H and Tubulin-βIII, but some negative cells were also observed (Figure 6d–f). Furthermore, there were Neurofilament-M-positive and MAP2-positive cells, and some double-positive cells were also observed. However, some cells were negative for both markers (Figure 6g–i). We therefore isolated the neural stem/progenitor cells cultured in the pre-differentiation medium by using a cell sorter with p75NTR as a marker (Figure 7a). Some cells were fixed for FCM analysis, and 67.7% of the p75NTR-positive cells (Figure 7b) were also Nestin-positive, so cells strongly positive for p75NTR were sorted (Figure 7c,d).

When cells that were strongly positive for p75NTR were sorted and cultured, they could be purified into cells that extended nerve cell-like processes (Figure 7e). In contrast, many p75NTR-negative and weakly positive cells showed a fibroblast-like morphology with a wide cytoplasm (Figure 7f). Each cell was cultured in a medium for differentiation into nerve cells for 7 days and cultured further for 7 days in a medium for differentiation into Recoverin-positive cells. When we determined the gene expressions of the cells immediately after isolation with p75NTR and those of the cells selected with p75NTR and differentiated into Recoverin-positive cells, we observed that the expression of the *Recoverin* gene was 36.6-fold higher in the cells differentiated from those strongly positive for p75NTR. However, the cells differentiated from cells that were weakly positive or negative for p75NTR had a 10.9-fold increased expression of the *Recoverin* gene. The sorting with p75NTR was able to concentrate cells with a *Recoverin* gene expression level approximately 3.4-fold higher than that of the unsorted cells (Figure 7g). In the cell immunostaining with Recoverin antibody, approximately 60% of cells were Recoverin-positive (Figure 7h).

### 3.5. Differentiation into Retinal Ganglion Cells

**Figure 8:**
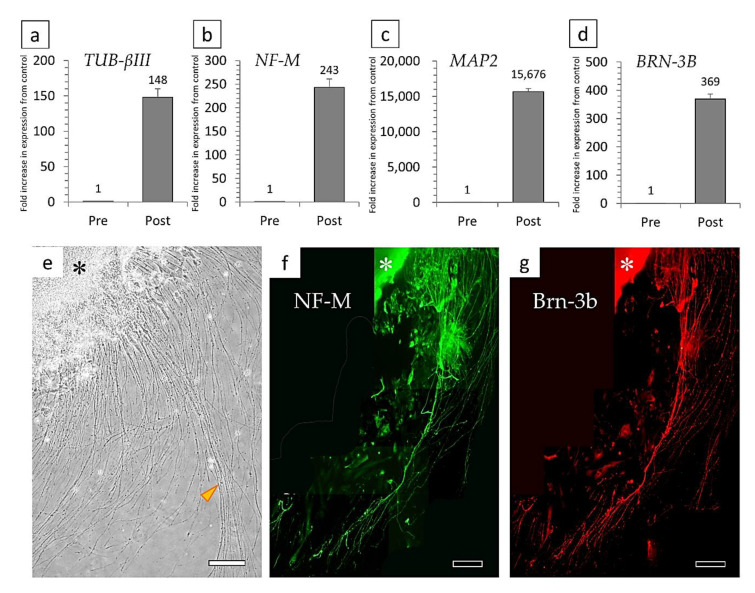
Relative semi-quantitative analysis of the gene expressions of (**a**) *TUBULIN-βIII*, (**b**) *NEUROFILAMENT-M*, (**c**) *MAP2*, and (**d**) *BRN-3B* in pre/post-differentiated cells. (**e**) An adhesive culture of cell aggregates (*) formed in suspension culture resulted in very long neurite outgrowth (*arrowhead*). (**f**,**g**) Fluorescent immunostaining results for markers of retinal ganglion cells: (**f**) Neurofilament-M, and (**g**) Brn-3b. Bars: 200 μm.

Differentiation into retinal ganglion cells was performed using the previously reported culture conditions and a method similar to that used for the differentiation of nerve cells [34]. p75NTR-positive cells were suspension-cultured for 7 days and then differentiated by adhesive culture. When the adhesive culture was performed, neurites began to elongate from the surface of the cell aggregates formed by the suspension culture (Figure 3d and Figure 8e). The cells that differentiated into ganglion cells had higher levels of *TUBULIN βIII* (148-fold increase), *NEUROFILAMENT-M* (243-fold increase), *MAP2* (15,676-fold increase), and the ganglion cell marker *BRN-3B* (369-fold increase) compared to before differentiation (Figure 8a–d). Cell immunostaining showed that the very long neurites were double-positive for Neurofilament-M and Brn-3b (Figure 8f,g).

### 3.6. Electrophysiological Recording

**Figure 9:**
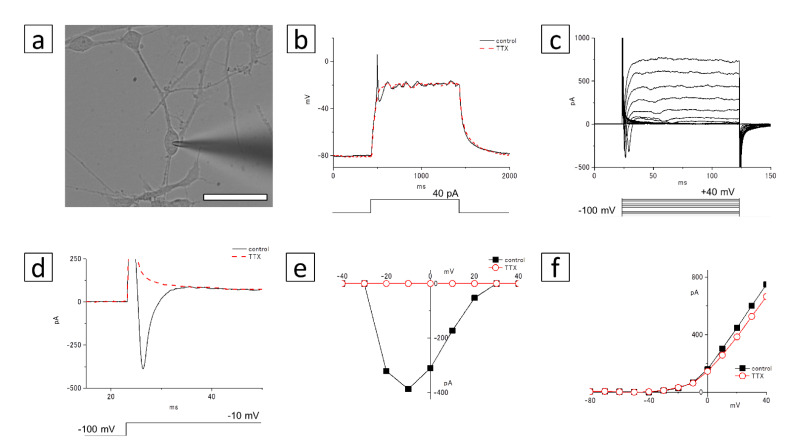
Patch-clamp recording of the Recoverin-positive cells. (**a**) A micrograph of the cultured cells. The patch electrode approaches the soma of the cell from the right side. Bar: 50 µm. (**b**) Responses to a depolarization induced by an injection of 40 pA current, recorded in control Ringer’s solution (*thin line*), and with the addition of 1 µM tetrodotoxin, a voltage-gated sodium channel blocker (TTX; *dotted line*). (**c**) Membrane currents of the cultured cells by depolarizing voltage steps from a holding potential of −100 mV. Command voltages were increased in 10 mV steps from −100 mV to +40 mV. (**d**) Command voltages were increased to −10 mV in control solution (*thin line*) or 1 µM TTX (*dotted line*). The inward current at the beginning of the voltage-step was blocked by TTX. (**e**) The I–V relationship of voltage-gated inward currents. The peak inward currents in control solution (*filled squares*) or in 1 μM TTX (*open circles*) were plotted against the test-pulse voltage. (**f**) The I–V relationship of voltage-gated outward currents. The amplitudes of outward currents in control solution (*filled squares*) or in 1 μM TTX (*open circles*) were plotted against the test-pulse voltage.

**Figure 10:**
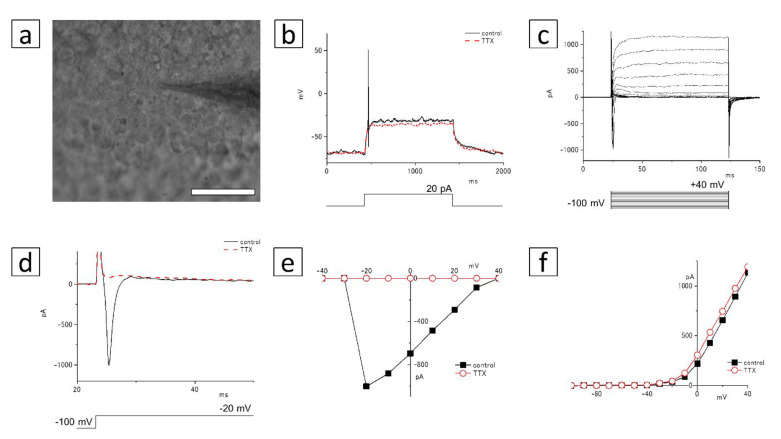
Patch-clamp recording of the Neurofilament-M- and Brn-3b-positive cells in the cell cluster. (**a**) A micrograph of the cultured cells. The patch electrode approaches the soma of the cell from the right side. Bar: 50 µm. (**b**) Responses to a depolarization induced by an injection of 20 pA current, recorded in control Ringer’s solution (*thin line*), and with the addition of 1 µM TTX (*dotted line*). (**c**) Membrane currents of the cultured cells by depolarizing voltage steps from a holding potential of –100 mV. Command voltages were increased in 10 mV steps from −100 mV to +40 mV. (**d**) Command voltages were increased to −20 mV in control solution (*thin line*) or 1 µM TTX (*dotted line*). The inward current at the beginning of the voltage-step was blocked by TTX. (**e**) The I–V relationship of voltage-gated inward currents. The peak inward currents in control solution (*filled squares*) or in 1 μM TTX (*open circles*) were plotted against the test-pulse voltage. (**f**) The I–V relationship of voltage-gated outward currents. The amplitudes of outward currents in control solution (*filled squares*) or in 1 μM TTX (*open circles*) were plotted against the test-pulse voltage.

To investigate whether the induced culture cells were differentiated into neurons, we measured the membrane voltage or current using the whole-cell patch clamp technique (Figure 9 and Figure 10). We first tested whether the Recoverin-positive cells can generate action potentials (Figure 9a). Under current-clamp conditions, a spike of the action potential was observed by a depolarizing current injection (+40 pA, Figure 9b). This spike was blocked by 1 µM tetrodotoxin (TTX, a voltage-gated sodium channel blocker; Figure 9b). To analyze the mechanism of this action potential, we measured membrane currents under voltage-clamp conditions (Figure 9c–f). As shown in Figure 9c, depolarizing voltage steps (from −100 to +40 mV) induced fast transient inward currents and delayed rectifier outward currents. This transient inward current disappeared with the addition of 1 µM TTX (Figure 9d). The TTX-sensitive inward currents began to be activated at −20 mV, and peaked at −10 mV (Figure 9e). The outward currents were not affected by 1 µM TTX (Figure 9f).

We next confirmed the response of the membrane voltage and current in the Neurofilament-M- and Brn-3b-positive cells (Figure 10a). The action potential was generated by a depolarizing current injection (+20 pA), and it disappeared after the addition of 1 µM TTX under current-clamp conditions (Figure 10b). Although the transient inward currents were observed with the depolarizing stimulations, they were blocked by TTX under the voltage-clamp conditions (Figure 10c,d). The peak amplitude of the inward current was recorded at −20 mV (Figure 10e). The delayed rectifier outward currents were detected with depolarizing stimulation at −30 to +40 mV (Figure 10f), and they were not affected by TTX.

The above-described results suggest that these culture cells differentiated into functional neurons. It was also revealed that these cells expressed voltage-gated sodium channels, which are involved in the generation of the action potential.

## 4. Discussion

Herein, H-iris iPS cells were created from H-iris stem/progenitor cells. Neural stem/progenitor cells sorted by p75NTR from these H-iris iPS cells could be differentiated into Recoverin-positive cells (photoreceptor-like cells) and retinal ganglion cells. These cells have been shown to function as nerve cells from the standpoint of electrophysiology.

The iPS cells were developed for the purpose of reprogramming differentiated human somatic cells and producing stem cells (pluripotent stem cells) that have pluripotency similar to ES cells. Unlike ES cells, iPS cells are created using their own somatic cells, thereby eliminating both the ethical issues and the issue of immune rejection. However, gene transfer is required to reprogram and produce iPS cells. It is expected that iPS cells can be used not only for cell transplantation therapy but also for medical research, drug discovery, the search for effective drugs, and for the prediction of the side effects of drugs. To actually apply iPS cells clinically, it is necessary to (1) establish a technique for more reliably differentiating only the target cells, and (2) determine the tumorigenesis of transplanted cells in detail. Here, we have demonstrated that a sorting method using p75NTR successfully selects cells with the potential to differentiate into more mature nerve cells while sorting out immature cells.

In various tissues, there are many differentiated cells to carry out specific functions of the tissue, and very few undifferentiated cells (tissue stem cells) exist. Tissue stem cells have the ability to replicate tissue stem cells that are identical to themselves (self-renewal ability) as well as the ability to differentiate into cells that form the tissue in which the tissue stem cells exist (differentiation ability). Some tissue stem cells were discovered that have the ability to differentiate into cells other than those of the tissues and organs in which the tissue stem cells existed [38,39,40]. With the use of tissue stem cells that are present in various tissues of the human body, it is expected that some of the problems associated with the use of ES cells and iPS cells will be minimized. However, it is necessary to consider issues such as efficient separation and maintenance culture methods for tissue stem cells from various tissues, and to establish a method for inducing reliable differentiation into target cells. We believe that regenerative medicine using tissue stem cells will be most useful for clinical applications. For that purpose, it is necessary to develop and establish a culture method and culture conditions that are capable of proliferating tissue stem cells in an undifferentiated state.

For iPS cells as well, before any future clinical applications can be pursued, we must first examine the effects of derived cells for producing iPS cells. It is also necessary to determine how to reprogram from differentiated somatic cells to the level of tissue stem cells rather than pluripotent stem cells. Kim et al. reported that the ability of iPS cells to develop in the three germ layers depends on the type of initiating cell, because iPS cells are affected during the reprogramming process by the epigenetic memory of the original cells [41]. Nishizawa et al. (2016) stated that the amount and pattern of DNA methylation in the process of reprogramming—rather than the particular cells from which iPS cells are derived—are important for cloning iPS cells that are more pluripotent and differentiated [42]. Reprogramming techniques and excellent clonal isolation have been reported to enable the generation of high quality iPS cells [43]. In order to produce iPS cells that can be more readily differentiated, it is necessary to continue research on the usability of the origin cells and on the effects of DNA methylation.

As a potential application of regenerative medicine research, a clinical study is underway to transplant a retinal pigment epithelial cell sheet derived from human iPS cells into patients with age-related macular degeneration [44]. Another study reported that retinal pigment epithelial cell sheets could be prepared in an automated closed culture system for regenerative medicine [45]. In our present investigation, the number of days required to differentiate from H-iris iPS cells to retinal pigment epithelial cells was half the number of days that was previously found to be required for the differentiation from fibroblasts to pigment epithelial cells (Supplementary Figure S1). Although this result alone is insufficient to prove the usefulness of H-iris iPS cells, it appears that H-iris iPS cells originally derived from melanin-bearing iris tissue cells may easily differentiate into melanin-expressing cells.

By using p75NTR to select cells during the process of neuronal differentiation, we were able to enrich electrophysiologically functional Recoverin-positive photoreceptor-like cells and retinal ganglion cells. For the differentiation and transplantation of iPS cell-derived cells in vitro, either the cells can be differentiated only to the target cells, or a method for eliminating undifferentiated cells is used. Here, we have devised a method for selecting cells that can differentiate into more mature neurons by pre-differentiating them to the stage of neural stem/progenitor cells and then sorting and concentrating them with p75NTR. As limitations of this novel method, we note that sorting with p75NTR alone cannot be selected only for highly differentiated cells, the intracellular signal may be changed by reacting the antibody with p75NTR, and the p75NTR antibody is completely not separated after sorting.

It was recently reported that the cells composing the eyeball continuously form layers on a culture dish and differentiate due to spontaneous contact between iPS cells [46]. The same study also mentioned that the cells differentiated in the same order as the cell layers that make up the eye, without any change in the condition of the medium. This is a phenomenon related to the differentiation mechanism of the new cells themselves, and further research is expected to provide more interesting information.

## 5. Conclusions

In conclusion, H-iris iPS cells were created from H-iris stem/progenitor cells. Then, by the sorting of neural stem/progenitor cells that were pre-differentiated from H-iris iPS cells with p75NTR, markers of retinal neurons were expressed, and we were able to concentrate Recoverin-positive cells (photoreceptor-like cells) with electrophysiological functions. By changing the culture method, retinal ganglion cells with electrophysiological functions were also differentiated. The series of results in this paper comprise the first findings in retinal nerve cell regeneration research.

## Figures and Tables

**Table 1:** Overview of ES and iPS cells.

Year	Person/Organization	Event
1981	Dr. M. Evans Dr. M. Kaufman	Created embryonic stem cells (ES cells) derived from the inner cell mass of mouse blastocysts [2]
1998	Prof. J. Thomson et al.	Successful production of human ES cells [3]
2006	Prof. S. Yamanaka et al.	Succeeded in producing iPS cells using skin cells of adult mice [4]
2007	Prof. S. Yamanaka et al.	Succeeded in producing iPS cells using human fibroblast cells [5]
2009	U.S. President B. Obama	Lifted the ban against the use of federal funding for human ES cell research

**Table 2:** Typical stem cells used in regenerative medicine research.

Cell Type	Cell Origin	Ethical Issues	Gene Transfer	Differentiation Ability
Embryonic stem (ES) cells	Fertilized egg (inner cell mass)	Yes	No	Versatile (every cell of adult tissue)
Induced pluripotent stem (iPS) cells	Body cells (autologous cells)	No	Yes	Versatile (every cell of adult tissue)
Somatic stem cells (tissue stem cells)	Body cells (autologous cells)	No	No	Limited (only limited cell types can be differentiated)

## Data Availability

All data was included in the main text and Supplementary Materials.

## Supplementary Materials

The following are available online at https://www.mdpi.com/article/10.3390/cells10040743/s1. Supplementary Figure S1: Outline of the differentiation of iPS cells into neural stem/progenitor cells, neural retina, and RPE. Supplementary Figure S2: Pigmented cells were observed at 30 days and were positive for RPE65 (a marker of retinal pigment epithelial cells) and ZO-1 (a marker of intercellular connections).
