# Supplementary material for: Novel Technique for Retinal Nerve Cell Regeneration with Electrophysiological Functions Using Human Iris-Derived iPS Cells
Source: Cells. 2021 Mar 28;10(4):743. doi: 10.3390/cells10040743 (PMC8067101; doi:10.3390/cells10040743)
Supplement: Supplementary file 1 [file cells-10-00743-s001.pdf]

cells

Article

Novel Technique for Retinal Nerve Cell Regeneration with Electrophysiological Functions Using Human Iris-Derived iPS Cells

Naoki Yamamoto [1,2,*], Noriko Hiramatsu [3], Mahito Ohkuma [4], Natsuko Hatsusaka [1], Shun Takeda [1], Noriaki Nagai [5], Ei-ichi Miyachi [4,6], Masashi Kondo [7], Kazuyoshi Imaizumi [7], Masayuki Horiguchi [8], Eri Kubo [1] and Hiroshi Sasaki [1]

Supplementary Materials:

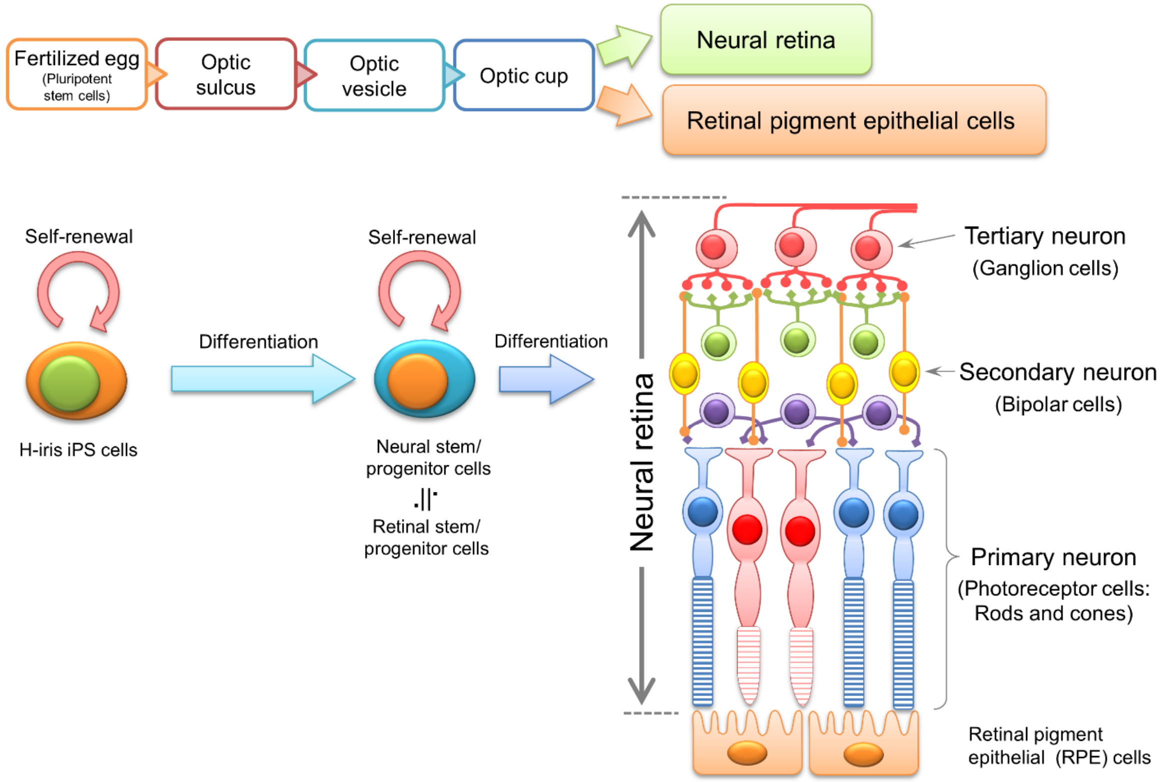

Figure S1. Outline of the differentiation of iPS cells into neural stem/progenitor cells, neural retina, and RPE.

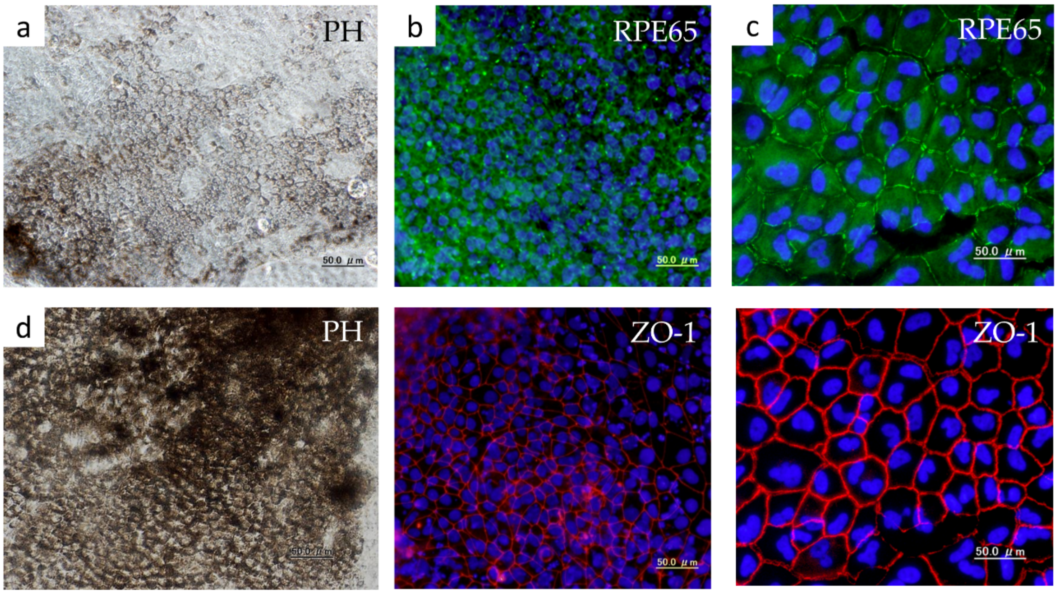

Figure S2. Pigmented cells were observed at 30 days and were positive for RPE65 (a marker of retinal pigment epithelial cells) and ZO-1 (a marker of intercellular connections).
